# Registered nurses’ exposure to high stress of conscience in long-term care

**DOI:** 10.1177/09697330231167542

**Published:** 2023-05-10

**Authors:** Hilde Munkeby, Grete Bratberg, Siri Andreassen Devik

**Affiliations:** Faculty of Nursing and Health Sciences, 158927 Nord University, Levanger, Norway; Centre of Care Research, Faculty of Nursing and Health Sciences, Nord University, Namsos, Norway

**Keywords:** Stress of conscience, nurses, nursing home, home nursing care, risk factors

## Abstract

**Background:**

In long-term care, registered nurses and other care providers often experience tensions between ideals and realities in the delivery of services, which can result in stress of conscience. Burnout, low quality of care and a tendency to leave the profession are perceived as consequences.

**Objectives:**

This study aimed to identify the socio-demographic and work-related factors associated with a high level of stress of conscience, particularly between nursing occupations.

**Research design:**

A cross-sectional survey was conducted among care providers who worked in Norwegian nursing homes and home care services in the spring of 2021. The sample consisted of 950 registered nurses and 1143 other care providers. Data were collected online using the Stress of Conscience Questionnaire (SCQ).

**Ethical considerations:**

Participation was voluntary and based on consent. The study was approved by the Norwegian Center for Research Data.

**Results:**

Registered nurses were nearly twice as likely to report high levels of stress of conscience compared to other care providers in long-term care. In addition, being a female, living alone, caring for their own children, working in an institution (versus home based), working >75% time, working shifts, not having scheduled meetings for ethical reflection and working in municipalities with a higher population density were factors associated with a high level of SCQ score.

**Discussion:**

Knowledge of factors that increase the risk of high SCQ scores in registered nurses provides opportunities for prevention**.** Managers in long-term care should pay more attention to how work is distributed between the occupational groups and should facilitate real opportunities for ethical reflection.

**Conclusions:**

The results of this study show that registered nurses have particular exposure to high levels of stress of conscience compared to other care providers in long-term care. Particular attention should be paid to registered nurses working in nursing homes.

## Introduction

In Scandinavia, the term ‘stress of conscience’ has emerged in recent decades, which is a concept that both differs from and extends the traditional understanding of moral distress.^1^ Based on an extensive literature review, Glasberg et al.^2^ identified situations that cause a ‘conscience that hurts’ in care providers: lacking time to provide care, being forced to provide care that feels wrong, dealing with incompatible demands, witnessing patients being offended, avoiding patients or family members, privacy affecting work, healthcare working conditions affecting time for self-care, not living up to others’ expectations and decreasing aspirations for good care. Hence, Glasberg et al.^2^ defined stress of conscience as stress generated by a troubled conscience that arises when one does not follow the voice of one’s conscience or when one’s personal conscience conflicts with oneself, others or society. Related concepts, such as moral distress and ethical distress, focus on the impact of dilemmas between right and wrong in clinical situations according to professional standards.^3^ Stress of conscience, on the other hand, does not focus on the ability or inability to take appropriate action but rather on the perception of conflict arising when being forced to negotiate the personal values that healthcare professionals hold.^1^ This study examines associations between a high level of stress of conscience and characteristics of different care providers across municipal care settings. In particular, attention is directed to registered nurses, who often by virtue of their education have overall professional responsibility and act as clinical leaders of care teams.

## Background

Long-term care in Norway is offered to a diverse group of people, from younger disabled people to the oldest and most frail patients.^4^ Services are provided in private homes and residential care homes, including nursing homes and assisted living facilities.^5^ However, changes in demographics and trends in healthcare services, such as increasingly individualised care and early discharge from hospital, challenge the organisation and allocation of services. In addition, a lack of competent employees and scarce financial resources make it difficult to offer services in line with new knowledge and intentions in the welfare state.^4,6^ Care providers, therefore, experience tension between the ideals and realities of how services are provided.^1,7,8^ The stress that this tension creates has been shown to threaten care providers’ personal and professional moral integrity, as well as their ability to act as moral agents.^1,9^

Examples of situations that contribute to stress of conscience in long-term care are the poor organisation of the working process, conflicting interpersonal relationships, difficulties with priorities and insufficient time to meet the patient’s needs.^10–12^ Burnout, low quality of care and a tendency to leave the profession are seen as consequences thereof^13,14^ and raise concerns within the care setting that is already challenged due to staffing issues.^15^ For instance, in 2018, Norwegian nursing homes and home care services reported a shortage of 8,000 registered nurses and other care providers, and since 2015, the shortage of registered nurses has doubled.^16^ Coinciding challenges are seen internationally.^17^ Due to their education (a 3-year bachelor’s degree), registered nurses have a special role and responsibility for those in the care system and are considered organisational and clinical leaders for the care teams of various care providers, both in Norway and internationally.^18,19^ Registered nurses have the responsibilities to lead the nursing team, supervise unskilled staff and allocate staff in line with patients’ care needs and staff skills and experience.^20^

In Norway, the care team mainly consists of auxiliary nurses (who have 2 years of upper secondary healthcare education and 2 years of experience as apprentices before obtaining a professional certificate) and nurse assistants (without formal healthcare education). Social educators (with a bachelor’s degree in healthcare) are also increasingly included in care teams.^21^ Social educators have knowledge and skills in health and care professions that give them the opportunity to work across agencies, borders and service user groups, including people with developmental disabilities.^22^ In Norway, social educators are often employed in the same positions as registered nurses, which means that they have the overall responsibility for nursing care, while other care providers meet patients’ immediate care and specific needs.^20^

Since stress of conscience is perceived as related to both internal and external demands,^2^ the phenomenon has been studied both from an individual and contextual perspective. Among contextual organisational factors, Åhlin et al.^14^ found that care providers in nursing homes report a higher degree of stress of conscience than care providers in home-based care, which the authors explained as being due to differences in the number of patients that an individual carer needs to care for at one time. Increased workload, in addition to organisational changes such as restructuring processes, has also been reported across environments and is linked to a higher degree of stress of conscience in general.^14,23,24^ Other contextual risk factors for the development of stress of conscience include a negative ethical climate, a lack of support from colleagues and management and limited opportunities to influence and discuss situations that create issues with conscience.^1,25,26^ However, factors in the organisation that are risk factors also have the potential to protect and prevent stress of conscience. A supportive environment and opportunities for reflection involving managers and employees, with the help of skilled personnel, have been shown to be important.^1,7^

On the individual level, socio-demographic variables, such as gender, age, marital status, childcare, education and work experience, have also been studied, resulting in varying findings.^1^ Åhlin et al.^27^ found that women report more stress of conscience than men, while Zhang et al.^28^ found no gender differences. Existing findings are especially contradictory in terms of age and work experience. While an older age appears to be a protective factor, stress of conscience increases with increasing work experience.^1,25^ Increased stress levels are also seen in care providers who work day shifts compared to those who only work night shifts.^29^ Moreover, the relationships among education, position and stress of conscience have been investigated, but no clear connections have been found or explained. For instance, some studies have found higher stress levels in registered nurses than other care providers with lower education,^30,31^ while others have identified high stress levels relating to differences in what triggers stress and how stress is perceived by or affects various care providers, regardless of education level.^7,26,32^

The knowledge base for this issue is weak, possible connections – whether individual or contextual – are unclear and no similar study has been carried out in Norway. This study is, therefore, considered important not only due to its clarification of the occurrence of stress of conscience in care providers and the identification of factors that might be influenced to ensure the recruitment and maintenance of registered nurses especially but also of other care providers, in the long-term care workforce.

## Aim

To identify socio-demographic and work-related factors associated with a high level of stress of conscience, in particular differences between nursing occupations.

## Methods

### Study design and participants

The study was designed as a cross-sectional survey, and the reporting is guided by the Consensus-Based Checklist for Reporting of Survey Studies (CROSS).^33^

We conducted the study in the municipal health service in two counties in Norway, which covers 79 municipalities with a total population of 700,000. The number of inhabitants in the municipalities varied from 450 to 205,000 and comprised both rural and urban areas. The study was conducted in the spring of 2021, when the COVID-19 pandemic was in its second year. During this period, the working situation for care providers in nursing homes and home care services was characterized by high work pressure, sick leave and changed working methods related to infection control, for example, visiting restrictions in nursing homes.^34,35^

A total of 10,154 care providers were asked to respond to an online survey between April and May 2021. Recruitment and the dispatch of questionnaires took place using the member registers at the Norwegian Nurses Organization (3,714 invited) and the Norwegian Union of Municipal and General Employees (6,440 invited). The survey was only sent to members who were employed in nursing homes and home care services, including residential care. In total, 2,132 participants consented to participate (a response rate of 21%). Registration options for profession were ‘registered nurse’, ‘social educator’, ‘auxiliary nurse’, ‘nurse assistant’, ‘apprentice’ and ‘other’. Participants who answered 'other' (39) were excluded, leaving a total sample of 2,093 care providers. The sample consisted of 925 registered nurses (43.4%), 25 social educators (1.2%), 980 auxiliary nurses (46%), 138 nurse assistants (6.5%) and 25 apprentices (1.2%).

### Survey instrument

The level of stress of conscience was estimated using the Stress of Conscience Questionnaire (SCQ) that Glasberg et al.^2^ developed. This instrument consists of nine items with two parts. In Part A, participants discuss how often they have experienced ethical stress in special situations using a six-point scale ranging from 0 = never to 5 = every day. In Part B, participants indicate the degree of stress that each situation causes. While the original instrument has a visual analogue scale divided into six points, we used tick options (six) from 0 = no, not at all to 5 = yes, gives me a lot of bad conscience. The scores from Part A and Part B are multiplied to reflect the score for each item, and the total level of stress of conscience is calculated by adding the sum for each item (maximum total score of 225).^2^ The higher the value, the higher the perceived stress level. A recent review^1^ has reported high internal consistency for this questionnaire, and in the present study, Cronbach’s alpha was 0.86 (95% confidence interval [CI]: 0.255–0.262). The instrument has also been validated in Nordic countries.^12,36^ The SCQ has been translated into Norwegian and used in a Norwegian study,^37^ but we found no studies in which the translation of the instrument into Norwegian or the validation of the Norwegian version has been published. Both forward and backward translations were conducted,^38^ and the Swedish researchers who originally developed the instrument were presented with the translation and gave their feedback. The instrument was also piloted in a sample of 10 healthcare workers. Both the linguistic and psychometric validation of the instrument will be published in a future article.

The survey comprised several socio-demographic and work-related questions, including self-reports about employees ethical working climate^32^ such as i) whether the manager facilitates ethical reflection (answers ranging from 0 = no, totally disagree to 5 = yes, totally agree) and ii) whether the department has scheduled meetings for ethical reflection (answers ranging from 0 = daily, to 5 = never).

### Data analysis

Statistical analysis was performed using the Statistical Package for the Social Sciences (IBM SPSS Statistics, version 28). The five original occupational groups were divided into two groups based on participants’ education level and task responsibility. In Group 1, we placed registered nurses and social educators, both of whom have health education at the bachelor’s level and responsibilities as the clinical leaders of nursing teams. Group 1 was labelled ‘Registered nurses’. Auxiliary nurses, nurse assistants and apprentices (who are students of practical studies before obtaining a certificate as auxiliary nurses) were placed in Group 2, labelled ‘Other care providers’. Descriptive frequency analyses were then used to investigate sample characteristics. The differences between the two occupational groups were tested using Chi-square tests for categorical variables and independent Student *t*-tests for continuous variables (Table 1). To identify and compare those with a higher SCQ score to the others, the sum score for the SCQ was recoded as a dichotomous variable (0 = low score, 1 = high score), with the cutoff for a high SCQ score set at ≥75%, that is, the highest quartile. Using multiple logistic regression analyses (Table 2), we investigated the crude and adjusted odds ratios (ORs) of reporting high SCQ scores associated with socio-demographic and work-related factors for the two occupational groups. We chose logistic regression with the estimation of the OR because this is a robust measurement method and, compared to linear regression, provides a more realistic and theoretically relevant treatment of a social phenomenon such as stress of conscience. Moreover, the SCQ instrument has been used in various countries with great variation in results, among samples and over time. This makes it difficult to compare results across countries and samples, especially based on mean scores (no limit values have previously been specified for what constitutes a little or a lot of stress of conscience.). Therefore, we chose to set the cutoff at the 75th percentile by using our own study population, not what is possibly normal, regardless of the pandemic or other conditions. The variables included in the logistic regression model were based on both theoretical assumptions and empirical findings.^1^

**Table 1.** Characteristics of the participants.

	Total	RNs^a^	OCPs^b^	
	*N* = 2093	*N* = 950	*N* = 1143	*p*-Value
Age, mean (SD)	45.2 (12)	43.4 (11.5)	46.7 (12.3)	<0.001
Missing	0	0	0	—
Gender	—	—	—	0.185
Female, *n* (%)	1953 (93.3)	894 (94.1)	1059 (92.7)	—
Male	140 (6.7)	56 (5.9)	84 (7.3)	—
Missing	0	0	0	—
Married/cohabiting, *n* (%)	1574 (75.2)	742 (78.1)	832 (72.8)	0.005
Missing	0	0	0	—
Caring for own children, *n* (%)	943 (45.1)	487 (51.3)	456 (39.9)	<0.001
Missing	0	0	0	—
Working municipality, > 10 000 inhbt, *n* (%)	1192 (57)	552 (58.1)	649 (56)	0.331
Missing	0	0	0	—
Main working place	—	—	—	<0.001
Nursing home, *n* (%)	1388 (66.3)	581 (61.2)	807 (70.6)	—
Homecare, *n* (%)	705 (33.7)	369 (38.8)	336 (29.4)	—
Missing	0	0	0	—
Special education, *n* (%)	701 (33.5)	384 (40.4)	317 (27.7)	<0.001
Missing	0	0	0	—
Working time fraction, > 75%, *n* (%)	1521 (72.7)	819 (86.2)	702 (61.4)	<0.001
Missing	0	0	0	—
Shift work	—	—	—	0.070
Daytime, *n* (%)	377 (18)	187 (19.7)	190 (16.6)	—
Shift, *n* (%)	1716 (82)	763 (80.3)	953 (83.4)	—
Missing	0	0	0	—
Years in main position	—	—	—	<0.001
<15 years, *n* (%)	1386 (66.2)	708 (74.5)	678 (59.3)	—
>15 years, *n* (%)	707 (33.8)	242 (25.5)	465 (40.7)	—
Missing	0	0	0	—
SCQ score, mean (SD)	77.1 (46.6)	87.4 (45.4)	68.5 (45.9)	<0.001
Missing	0	0	0	—
SCQ, high score ≥75 pct, *n* (%)	536 (25.6)	307 (32.3)	229 (20)	<0.001
Missing	0	0	0	—
Leader facilitates ethical reflection	—	—	—	<0.001
Largely or fully agree, *n* (%)	555 (26.5)	212 (22.3)	343 (30)	—
Missing	12 (0.6)	4 (0.4)	8 (0.7)	—
Scheduled meetings for ethical reflection	—	—	—	<0.001
Regularly, *n* (%)	432 (20.6)	151 (15.9)	280 (24.5)	—
Seldom/never, *n* (%)	1612 (77)	788 (82.9)	824 (72.1)	—
Missing	50 (2.4)	11 (1.2)	39 (3.4)	—

^a^RNs, registered nurses.

^b^OCPs, other care providers.

**Table 2.** Likelihood of reporting a high SCQ score associated with nursing occupation, socio-demographic and work-related conditions.

		Unadjusted OR	95% CI	Adjusted OR^a^	95% CI	*p*-Value
Nursing occupation	OCP^b^	—	—	—	—	—
—	RN^c^	1.91	1.56–2.32	1.73	1.39–2.16	<0.001
Socio-demographic characteristics						
Age	Year	0.98	0.98–0.99	0.99	0.98–1.00	0.061
Gender	Male	—	—	—	—	—
	Female	3.91	2.15–7.13	3.76	2.04–6.92	<0.001
Married/cohabiting	Yes	—	—	—	—	—
	No	1.14	0.91–1.43	1.31	1.03–1.67	0.026
Caring for children	Yes	—	—	—	—	—
	No	0.74	0.60–0.89	0.79	0.64–0.99	0.037
Special education	Yes	—	—	—	—	—
	No	1.05	0.85–1.20	1.07	0.85–1.34	0.582
Work-related conditions						
Working municipality	≤10,000	—	—	—	—	—
	>10,000	1.38	1.12–1.68	1.33	1.08–1.64	0.008
Main working place	Homecare	—	—	—	—	—
	Nursing home	1.30	1.05–1.60	1.41	1.13–1.77	0.002
Working time fraction	≤75%	—	—	—	—	—
	>75%	1.71	1.35–2.17	1.27	0.98–1.65	0.072
Working shifts^d^	Yes	—	—	—	—	—
	No	0.45	0.33–0.61	0.47	0.34–0.64	<0.001
Scheduled meetings for ethical reflection^e^	Yes	—	—	—	—	—
	No	1.86	1.42–2.44	1.74	1.32–2.31	<0.001

Hosmer–Lemeshow test: Chi-square = 3.267, df = 8, *p* = 0.92.

^a^Adjusted OR shows the OR adjusted for all other variables in the model.

^b^OCPs, other care providers.

^c^RNs, registered nurses.

^d^Working 2 or 3 shift.

^e^Every month or more often.

Sample differences between the two occupational groups related to age, marital status, childcare and others (Table 1) constituted both variables of interest as well as confounders and were therefore adjusted for in the multivariable model. However, the variable ‘years in main position’ was considered not to provide valid information about work experience (i.e. it is possible to have spent a short time in one’s main position but a long time in previous positions) and was thus omitted from the model. All independent variables, except ‘age’, were dichotomised and used as dummies. Due to collinearity, only one of the two ethical working climate variables (‘Scheduled meetings for ethical reflection’) was included in the model. Additionally, the variable ‘workplace’ was divided into ‘home-based care’ and ‘nursing homes’ (which included residential care because this context in practice functions as institution-based care). Estimates were also presented with 95% CIs. The Hosmer–Lemeshow test was then used for the goodness of fit of the statistical model, and *p-*values of less than 0.05 were considered statistically significant.

Finally, SCQ item-specific differences (including responses to Part A and Part B, separately) between the two occupational groups were tested using independent sample *t*-tests and reported as mean differences with CIs (Table 3).

**Table 3.** Item-specific differences in mean SCQ scores between registered nurses (RNs) and other care providers (OCPs).

SCQ index item	Mean (SD) RN	Mean (SD) OCP	Mean differences	CI (lower–upper)	
1. How often do you lack time to provide the care the patient needs?	14.3 (6.9)	13.6 (7.7)	−0.68	−1.31	−0.48
A. Frequency	3.6 (1.3)	3.3 (1.5)	−0.31	−0.43	−0.19
B. Degree of stress of conscience	3.8 (1.3)	3.8 (1.5)	0.03	−0.09	0.15
2. Are you ever forced to provide care that feels wrong?	10.0 (7.2)	8.9 (7.9)	−1.12	−1.77	−0.47
A. Frequency	2.5 (1.5)	2.1 (1.7)	−0.34	−0.47	−0.20
B. Degree of stress of conscience	3.4 (1.7)	3.0 (2.0)	−0.37	−0.53	−0.21
3. Do you ever have to deal with incompatible demands in your work?	11.8 (7.3)	8.4 (7.5)	−3.37	−4.02	−2.73
A. Frequency	3.1 (1.4)	2.2 (1.6)	−0.85	−0.98	−0.72
B. Degree of stress of conscience	3.4 (1.5)	2.8 (1.9)	−0.60	−0.74	−0.45
4. Do you ever see patients being insulted and/or injured?	7.4 (6.7)	6.4 (7.1)	−0.99	−1.58	−0.40
A. Frequency	1.8 (1.4)	1.5 (1.5)	−0.30	−0.42	−0.17
B. Degree of stress of conscience	3.2 (2.0)	2.8 (2.2)	−0.44	−0.61	−0.26
5. Do you ever find yourself avoiding patients or family member who need help or support?	6.1 (6.7)	3.5 (5.9)	−2.65	−3.19	−2.10
A. Frequency	1.5 (1.4)	0.9 (1.3)	−0.64	−0.76	−0.52
B. Degree of stress of conscience	2.6 (2.1)	1.8 (2.1)	−0.82	−1.00	−0.64
6. Is your private life ever so demanding that you do not have the energy to devote yourself to your work as you would like?	5.7 (6.4)	4.1 (5.8)	−1.63	−2.16	−1.11
A. Frequency	1.6 (1.4)	1.1 (1.3)	−0.49	−0.60	−0.37
B. Degree of stress of conscience	2.5 (1.9)	2.0 (2.1)	−0.46	−0.63	−0.29
7. Is your work in healthcare ever so demanding that you do not have the energy to devote yourself to your family as you would like?	13.2 (7.6)	10.3 (8.1)	−2.97	−3.64	−2.29
A. Frequency	3.0 (1.4)	2.4 (1.6)	−0.63	−0.76	−0.51
B. Degree of stress of conscience	3.8 (1.6)	3.3 (1.9)	−0.54	−0.69	−0.39
8. Do you ever feel that you cannot live up to others` expectations of your work?	9.1 (7.4)	6.6 (7.2)	0.2.52	−3.15	−1.89
A. Frequency	2.5 (1.4)	1.8 (1.5)	−0.66	−0.79	−0.54
B. Degree of stress of conscience	3.0 (1.7)	2.5 (1.9)	−0.57	−0.73	−0.42
9. Do you ever lower your aspirations to provide good care?	9.8 (7.6)	6.8 (7.5)	−2.95	−3.60	−2.30
A. Frequency	2.5 (1.5)	1.8 (1.6)	−0.68	−0.82	−0.54
B. Degree of stress of conscience	3.2 (1.8)	2.5 (2.0)	−0.76	−0.93	−0.60

Mean values (m), standard deviations (SD), mean differences and CIs.

### Ethical considerations

Approval of the project was granted by the Norwegian Centre for Research Data (project no. 571318). The participants were informed and invited to the survey via email, mediated via the trade unions. It was pointed out that the research did not take place under the auspices of the trade union but the research group. The research group had no access to the email addresses. The participants actively consented by ticking a box in the survey.

## Results

### Demographics

Socio-demographic and work-related characteristics are shown in Table 1. Registered nurses were younger than other care providers (mean = 43.4 versus mean = 46.7 years, CI: 2.26–4.31), more likely to be married (or cohabiting) (*X*^*2*^ = 7.86, *phi* = −0.06) and also caring for their own children (*X*^*2*^ = 27.08, *phi* = −0.11). Working in nursing homes was more common for other care providers, while more registered nurses (39%) than other care providers (30%) worked in home-based care (*X*^*2*^ = 53.10, *phi* = 0.16). A higher proportion of other care providers (41%) than registered nurses (26%) had been working in the same position for more than 15 years (*X*^*2*^ = 53.65, *phi* = −0.16), while the current working time fraction (>75% position) was higher among registered nurses (86%) than other care providers (61%) (*X*^*2*^ = 160.58, *phi* = 0.28). Special education (e.g. in geriatrics) was more common in registered nurses (40%) than in other care providers (28%) (*X*^*2*^ = 37.49, *phi* = −0.13).

The mean SCQ score was higher in registered nurses than in other care providers and therefore a higher reported SCQ score (≥75 percentile) was more common in registered nurses (32%) than in other care providers (20%) (CI: −0.16 to −0.09). Mean SCQ scores increased with higher levels of nursing education. Registered nurses reported a higher score on average than all other groups of care providers (*p* < 0.001) (the difference between registered nurses and social educators was not statistically significant). Further analyses and comparisons were made between care providers with a bachelor’s degree (registered nurses *n* = 925) versus those with lower or no formal education (other care providers *n* = 1143).

A higher percentage of other care providers (25%) than registered nurses (16%) reported having regularly scheduled meetings for ethical reflection (*X*^*2*^ = 26.26, *phi* = 0.11), and compared to registered nurses (22%), other care providers (30%) were also more likely to largely or fully agree that their leaders facilitated for ethical reflection (*X*^*2*^ = 16.10, *phi* = −0.09).

### Factors associated with high level of stress of conscience and differences related to occupation

Given that a higher level of stress of conscience constitutes a risk factor, those with sum scores above the third quartile (≥75 percentile) in this study sample were classified as having high levels of stress of conscience. Table 2 shows the associations between socio-demographic and work-related factors and high score of stress of conscience as the outcome variable reported as unadjusted and multivariable-adjusted ORs with 95% CIs.

Adjusted for all individual and work-related factors included in the model, registered nurses were more likely to report a high score of stress of conscience compared to other care providers (OR: 1.73, 95% CI: 1.38–2.16). In addition, being a female, caring for their own children, single living, working in an institution (versus home based), working >75% time, working shifts, not having scheduled meetings for ethical reflection and working in municipalities with higher population density were independently of all other factors, associated with a high level of SCQ score.

### Stress of conscience and item-specific differences between registered nurses and other care providers

To gain greater knowledge about the differences between the two occupational groups regarding stress of conscience, each of the nine items was analysed separately. Table 3 shows item-specific differences in mean SCQ scores between registered nurses and other care providers.

The means varied largely between SCQ items but with very similar patterns of reporting in the two occupational groups. The first item about lacking time to provide needed care received the highest mean score in both registered nurses (mean = 14.3) and other care providers (mean = 13.6) and was the item that showed the lowest mean difference between the two groups (mean difference = −0.68) (Table 3). The item that differed mostly between the two occupational groups was ‘Do you ever have to deal with incompatible demands in your work’ (item 3) followed by ‘Is your work in healthcare ever so demanding that you do not have the energy to devote yourself to your family as you would like’ (item 7) and ‘Do you ever lower your aspirations to provide good care’ (item 9). Registered nurses reported higher mean scores on all nine SCQ items than other care providers.

The variations in the mean difference between the groups showed no clear pattern. Both groups reported varyingly how often they experienced given situations and the degree of stress this entailed. Regarding item 3, where the mean difference was greatest, it was clear that registered nurses reported being in situations involving incompatible demands more often than other care providers.

## Discussion

Findings from the multivariable logistic regression analysis showed that registered nurses were nearly twice as likely to report high levels of stress of conscience compared to other care providers in long-term care. In comparison with other studies from similar contexts, the mean score of SCQ for registered nurses was higher in our study (87.4) than in previous studies, where average scores varied from 35 to 64 regardless of occupational groups.^1^ The survey was conducted during the COVID-19 pandemic, which may explain the findings of a higher level of stress of conscience than in previous studies, due to more work and concerns about infection management. Registered nurses experienced that the pandemic led to a higher workload, reduced job satisfaction and major ethical challenges in Norwegian long-term care.^34,35^ However, studies carried out during the pandemic in hospitals show lower scores on stress of conscience than in previous studies carried out in municipal elderly care before the pandemic.^39^ It can therefore be difficult to explain in what way the pandemic affected the experience of stress of conscience.

Swedish studies have shown that registered nurses score higher on stress of conscience than other care providers in elderly residential care,^7^ but not in psychiatric care,^31^ which may indicate that the care context plays a role. In the present study, care providers who worked in nursing homes were 1.4 times more likely to report high SCQ scores than those who worked in home-based care. The result can be attributed to various explanations.

The patients in nursing homes are the oldest and most vulnerable and, unlike home nursing care, the service tasks are not specified in advance.^40^ The absence of pre-defined tasks can provide a greater opportunity for person-centred care, which has been shown to counteract stress of conscience.^3,41^ However, this requires enough time to carry out the care in line with the intentions. Since item 1 (How often do you lack time to provide the care that the patient needs) contributed to high scores on SCQ, we assume that time was experienced as insufficient. In any case, the care tasks in a nursing home can be experienced as limitless.^8^ In our study, there were also significantly more registered nurses working in home nursing care than nursing homes, which indicates fewer registered nurses take responsibility for nursing tasks in nursing homes.

The documented shortage of registered nurses^42^ means a greater workload where different tasks are set against each other, and registered nurses have to prioritise between more or less acute tasks. In such a situation, care tasks can be downgraded and patients may not have their social or existential needs met.^43^ This may explain why item 3 (Do you ever have to deal with incompatible demands in your work) showed the greatest mean difference in SCQ score between registered nurses and other care providers. In our sample, the difference was particularly about registered nurses more often experiencing situations with incompatible demands than other care providers (cf. Table 3). This is a difference supported by Juthberg et al.,^7^ who found that registered nurses exhibited sensitivity to expectations and demands in their work. Johansen and Fagertsröm^44^ suggest that registered nurses in home-based care must direct their attention to those who need nursing skills the most and that activities should be better allocated between registered nurses, social educators, assistant nurses and informal caregivers. Karlsson et al.^45^ highlighted that registered nurses’ role in municipal elderly care in Sweden entailed paradoxical expectations, where registered nurses should be leaders, but simultaneously equal and subordinate. Juthberg et al.^7^ perceive a need among professional groups to gain greater insight into both their own and each other’s occupational situations. Studies also show that registered nurses have little opportunity to develop confidence and competence as leaders and lack guidance for this work in elderly care and may thus be unprepared for the complex role they play.^20,46^

Care providers who reported not having scheduled meetings for ethical reflection were nearly twice as likely to report high SCQ scores as those who reported having the offer. Previous studies have highlighted the importance of an arena for reflection, where employees can express their guilty conscience.^1,2,29^ Reflection is considered the most important means of developing moral competence and has contributed to employees being more aware of ethical issues in their work.^47,48^ Discussing ethical challenges with colleagues also provides an opportunity to explore the problems in-depth, find solutions together and emotionally support each other.^8,49,50^ In previous studies, social support has been shown to be protective against the development of stress of conscience.^14^ In our sample, significantly more other care providers compared to registered nurses answered that they had planned ethical reflection meetings. The causes of this are unknown but should be investigated further. However, ethical reflection can be de-prioritised in hectic work periods, and there are also different understandings of what ethical reflection is and should contain.^51^ For example, reporting situations or other professional meetings can be perceived by some as ethical reflection.^51^ If registered nurses do not experience these meetings, they risk losing opportunities to discuss which premises the work should be based on which may weaken their ability to shape the ethical climate in the department and influence the quality of the work they lead. Registered nurses in this sample scored relatively higher than other care providers on item 9 which concerned stress of conscience associated with lowering their ambitions for good care. Qualitative findings support the connection between nurses’ high ideals for care (internal demands) and the development of troubled conscience when the goals cannot be achieved.^8^

Among the socio-demographic factors associated with reporting a high SCQ score, women were almost four times more likely to score high on stress than men, which is consistent with previous findings.^1,14^ Age had little impact on the likelihood of reporting a high SCQ score in our study. Single care providers were 1.7 times more likely to report a high score than those who were cohabiting. Furthermore, being childless appeared to be a slightly protective factor. This result contrasts the findings in the review by Jokwiro et al.,^1^ which found that being married and childless appeared as risk factors. The registered nurses in our sample were younger and had more caring responsibilities for children than other care providers, which may explain the high reporting of conscience stress on item 7: ‘Is your work in healthcare ever so demanding that you do not have the energy to devote yourself to your family as you would like’. Working in municipalities with a population density of less than 10,000 also emerged as a protective factor in our sample. Research shows that a phenomenon such as missed care, which in turn increases the risk of moral stress among care providers, can be linked to organisational conditions such as staffing, workload, funding and leadership.^52^ Norwegian municipalities show great variation when it comes to the quality of health and care services.^53^ Possible connections between municipality size and work-related conditions for care providers that increase the risk of stress of conscience should be investigated further.

### Strengths and limitations

Although the response rate in this study was 21%, the trade unions’ overview of socio-demographic and work-related factors (sex, age and further education) showed that our sample is representative of the target population of the survey.

The Norwegian version of the SCQ has not been linguistically and culturally validated. However, there are linguistic similarities between the Norwegian and Swedish languages and the countries are also largely culturally comparable. The questionnaire has been validated in Sweden and other Nordic countries, and Cronbach’s alpha in the present study corresponds to previous studies.

While the original SCQ uses a visual analogue scale on the B part, in this study we used a six-point scale, which may complicate the comparison of our results with other studies. However, other studies have shown an agreement between visual analogue scale and numeric scale which may indicate that a comparison of findings is credible.^54^ Furthermore, it can be questioned whether the phenomenon of stress of conscience is measured more or less accurately with a decimal level than a point scale and whether it is crucial to get a realistic estimate. There is no stated cutoff for high/low sum scores in the SCQ. Our cutoff for a high score (75% percentile) was based on calculations about the normal distribution in our sample. This limit is statistical and does not provide any information about the possible clinical significance of high/low scores on stress of conscience. We acknowledge that our variable ‘years in main position’ was too imprecise to be used in the analysis. Rather, we should have asked a question about ‘years in the profession’ to possibly investigate the connections between age and work experience. The survey was designed so that the participants had to answer each question to continue and complete the questionnaire. We had no missing values on SCQ.

## Implications

The results give reason to further investigate registered nurses’ role in both nursing homes and home nursing care, particularly their role as a professional leader and how this affects and is affected by stress of conscience. Educational institutions must also prepare nursing students on how to handle demands that may contribute to a guilty conscience after graduation. Managers should pay increased attention to how work is distributed between occupational groups and facilitate a real opportunity for participation in reflection where the factors that contribute to a troubled conscience are openly discussed.

## Conclusion

The results of this study show that registered nurses have particular exposure to high levels of stress of conscience compared to other care providers in long-term care. In addition, we found several other factors that independently increased the risk of high stress levels but which could also act as protective factors. The results can be used to draw attention to work-related factors that give registered nurses stress of conscience in both nursing homes and homecare services. Knowledge of protective factors such as facilitating ethical reflection is particularly relevant for managers. It is also a managerial responsibility to put role clarification and division of tasks between employees on the agenda, especially at a time when there is a shortage of registered nurses in Norway and other countries. We perceive particular cause for concern for registered nurses working in nursing homes.
